# Electronic properties of the boroxine–gold interface: evidence of ultra-fast charge delocalization†
†Electronic supplementary information (ESI) available: Details about the calculations, further theoretical results, STM images of the thermally annealed TPB monolayer and XPS data analysis procedure are reported. See DOI: 10.1039/c6sc05632f



**DOI:** 10.1039/c6sc05632f

**Published:** 2017-03-08

**Authors:** Daniele Toffoli, Matus Stredansky, Zhijing Feng, Gabriele Balducci, Sara Furlan, Mauro Stener, Hande Ustunel, Dean Cvetko, Gregor Kladnik, Alberto Morgante, Alberto Verdini, Carlo Dri, Giovanni Comelli, Giovanna Fronzoni, Albano Cossaro

**Affiliations:** a Department of Physics , University of Trieste , via A. Valerio 2 , 34127 , Trieste , Italy; b CNR-IOM Laboratorio Nazionale TASC , Basovizza SS-14, km 163.5 , 34012 Trieste , Italy . Email: cossaro@iom.cnr.it; c Department of Chemical and Pharmaceutical Sciences , University of Trieste , via L. Giorgieri 1 , 34127 , Trieste , Italy . Email: fronzoni@units.it; d Faculty of Mathematics and Physics , University of Ljubljana , Jadranska 19 , Ljubljana , Slovenia; e J. Stefan Institute , Jamova 39, SI-1000 , Ljubljana , Slovenia; f Department of Physics , Middle East Technical University , 06531 Ankara , Turkey

## Abstract

We performed a combined experimental and theoretical study of the assembly of phenylboronic acid on the Au(111) surface, which is found to lead to the formation of triphenylboroxines by spontaneous condensation of trimers of molecules. The interface between the boroxine group and the gold surface has been characterized in terms of its electronic properties, revealing the existence of an ultra-fast charge delocalization channel in the proximity of the oxygen atoms of the heterocyclic group. More specifically, the DFT calculations show the presence of an unoccupied electronic state localized on both the oxygen atoms of the adsorbed triphenylboroxine and the gold atoms of the topmost layer. By means of resonant Auger electron spectroscopy, we demonstrate that this interface state represents an ultra-fast charge delocalization channel. Boroxine groups are among the most widely adopted building blocks in the synthesis of covalent organic frameworks on surfaces. Our findings indicate that such systems, typically employed as templates for the growth of organic films, can also act as active interlayers that provide an efficient electronic transport channel bridging the inorganic substrate and organic overlayer.

## Abstract 2

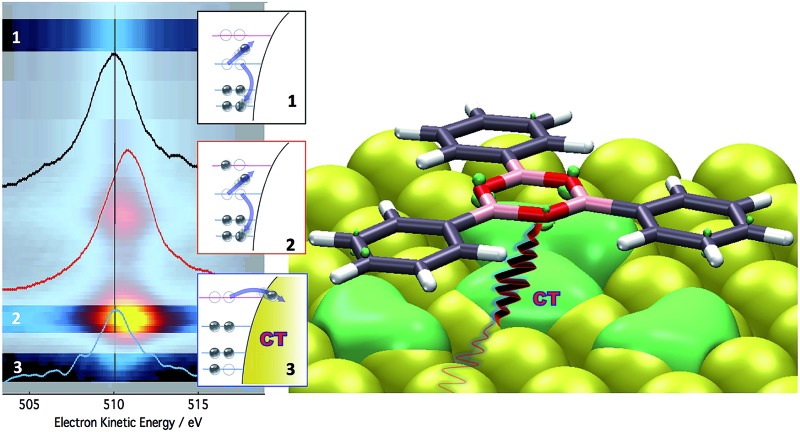
A combined theoretical and experimental study of the assembly of triphenylboroxines on Au(111) reveals the charge delocalization properties of the interface.

## 1 Introduction

**Fig. 1:**
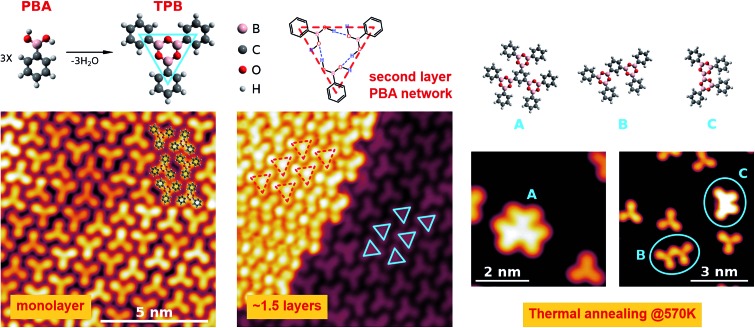
Top panel: Schematic representation of the PBA and TPB molecules (left) and of the suggested hydrogen bonding network of PBA trimers forming the second molecular layer (center). Models on the right (A–C) represent possible configurations for the polymerized structures obtained upon thermal annealing at 570 K. Bottom panels: STM images of the monolayer (left), of the 1.5 layer (center) and of the sub-monolayer obtained upon thermal annealing at 570 K (right). Blue and red triangles indicate the size and shape of the TPB molecule and PBA trimers forming the first and second layer, respectively. STM images parameters: left, *V*_s_ = –2.0 V, *I*_t_ = 0.5 nA, 10 × 10 nm^2^; center, *V*_s_ = +0.1 V, *I*_t_ = 0.1 nA, 10 × 10 nm^2^; right small images: *V*_s_ = +0.1 V, *I*_t_ = 0.5 nA, 5 × 5 nm^2^, and *V*_s_ = –2 V, *I*_t_ = 0.2 nA, 7 × 7 nm^2^, respectively.

Boronic acids represent versatile and widely adopted building blocks for organic synthesis and for the formation of organometallic compounds.^1^ Among the different processes they are involved in, boronic terminations can undergo a condensation process when interacting with diols or upon their auto-recognition process, leading to the formation of covalent intermolecular networks.^2–6^ This aspect has become a topic of interest in surface chemistry, where the possibility to tailor the structural and chemical properties of the substrates at the nanoscale is crucial, with the aim of building complex organometallic interfaces with suitable transport properties for organic electronics.^7–10^ In this view, boronic acids are exploited in the synthesis of 2D Covalent Organic Frameworks (COFs) as templates on metal surfaces^11,12^ as well as of catalytically inactive graphite^13–15^ and graphene.^16^ The perspective role of these systems in 2D guest–host chemistry has been recently demonstrated with the assembly of fullerenes on a boronic COF grown on a graphite surface.^17^ Even though the synthesis of boronic COFs is extremely efficient both in solution and under Ultra High Vacuum (UHV) conditions, the morphology of the resulting template differs from an ideal honeycomb due to the proliferation of defects during the condensation process.^18^ Concerning the growth in solution, a valuable route to obtain more regular structures relies on the reversibility of the condensation process. In the case of auto-condensation, the exposure of the system to H_2_O promotes the equilibrium in the boroxine formation reaction and leads to COFs of better quality.^19^ On the other hand, for systems grown under UHV, the morphology of the COFs was shown to strongly depend on the molecule–substrate interaction and on the growth conditions.^20^ In addition to the morphological defects, another aspect limiting the use of boroxine-based templates lies in their electronic transport properties. In fact, boroxine groups possess a low aromaticity, which prevents the formation of dispersive electronic band structures close to the Fermi level.^21^ Unlike other 2D materials like graphene or boron nitride, only poor electronic transport can be therefore expected for the boroxine-based COFs. On the other hand, boroxine systems show better performance in terms of their response to mechanical strain.^21^ These considerations suggest that although boroxine-based 2D systems cannot be directly employed as electrodes, they can nevertheless serve as templates for the growth of complex organic architectures, being particularly suitable for flexible electronics thanks to their mechanical properties. In this context it becomes relevant to evaluate if they can also tailor the electronic properties of the electrodes. To this aim, a spectroscopic study of the boroxine–metal interface becomes mandatory, possibly providing relevant information towards an improvement of the morphologic properties of the systems. In this view, here we present a combined experimental and theoretical study of a simple boronic molecule, namely phenylboronic acid (PBA, C_6_H_7_BO_2_, reported in the upper panel of Fig. 1), undergoing auto-condensation on the Au(111) surface. To our knowledge, the characterization of the assembly of PBA ultra-thin films has not been reported yet, possibly because of the limited interest for this molecule in the synthesis of COFs. In fact, PBA has only one boronic group, thus preventing the formation of extended covalent structures in the condensed phase. The molecule nevertheless represents a suitable reference for studying the electronic properties of the boroxine group. In fact, the condensation of three PBA molecules leads to the formation of a triphenylboroxine (TPB, shown in the upper panel of Fig. 1), whose simple structure can be easily studied both experimentally and theoretically. In the following paragraphs we report complete characterization of both PBA and TPB films by means of X-ray Photoemission Spectroscopy (XPS), X-ray Absorption Spectroscopy (NEXAFS) and Scanning Tunneling Microscopy (STM), providing a detailed description of the chemistry and morphology of the systems. Density Functional Theory (DFT) calculations and Resonant Auger Electron Spectroscopy (RAES) are eventually employed to study the electronic coupling and, in particular, the charge delocalization dynamics at the organo-metallic interface. Most of the previous studies on boroxine COFs were focused on the study of the morphology by means of STM. Taking a significant step further, our study now adds a reference for the electronic properties of these and similar systems, in particular for the electronic coupling of the unoccupied Molecular Orbitals (MOs). More interestingly, the results we report highlight promising properties of the boroxine group in terms of bridging the charge transport between the substrate and the organic overlayer, which is a key issue for an effective implementation of these systems in organic electronics.

## 2 Methods

### 2.1 System preparation

Au(111) single crystal was cleaned by Ar^+^ sputtering and annealing cycles. PBA (Sigma Aldrich, 97%) was evaporated from a Pyrex vial connected to the experimental chamber through a leak valve. A 5 × 10^–7^ mbar, 5 minutes long exposure was necessary to obtain monolayer saturation, with the sample kept at room temperature. The formation of a multilayer was observed for depositions at sample temperatures *T*_s_ < 230 K.

### 2.2 Experimental details

The X-ray spectroscopy measurements were performed at the ALOISA beamline of the Elettra laboratory.^22^ XPS measurements were performed at a photon energy of 650 eV, with an overall resolution of 300 meV. The Au 4f_7/2_ peak (B.E. 84.0 eV (ref. 23)) was measured for each scan to calibrate the binding energy scale. The stoichiometry of the films was analyzed by taking into account the cross sections of the different elements (*σ*_Au 4f_ = 2.21 Mbarn, *σ*_O 1s_ = 0.338 Mbarn, *σ*_C 1s_ = 0.136 Mbarn and *σ*_B 1s_ = 0.071 Mbarn (ref. 24)). In the case of the thick film, the different inelastic mean free paths (IMFP)^25^ of the electrons have been considered in order to both determine the thickness of the film and properly normalize the intensities of the different peaks. The thickness of the thick film has been estimated to be 55 ± 10 Å.

O and B K-edge NEXAFS spectra were acquired in partial electron yield, with a cutoff of the secondary electrons at 480 eV and 150 eV, respectively, and with an overall resolution of 200 meV and 100 meV, respectively. The RAES map was acquired under normal emission and grazing incidence conditions, with the polarization of the electric field oriented 4 deg from the surface normal. STM imaging was performed with an Omicron Low Temperature STM, hosted in a custom-built experimental UHV system, operating at a base pressure of 1 × 10^–10^ mbar. Electrochemically etched tungsten tips were used for imaging. Images were acquired in constant current mode, at a temperature of approximately 5 K; the applied sample bias with respect to the grounded tip is reported in the images; a positive bias thus indicates imaging of the sample empty states. Ball models are rendered with the XCrySDen, Avogadro, and VMD software packages.^26–28^

### 2.3 Theoretical methods

A two-step computational scheme has been followed. In the first step a periodic slab methodology was employed for the geometry optimization of the adsorption models while in the second step suitable finite clusters were cut from the periodic relaxed structures and used for the calculation of core excitation energies and oscillator strengths of the adsorbed molecule. The periodic DFT calculations were performed with the Kohn–Sham orbitals expanded in a plane wave basis and electron–nuclear interaction described by ultrasoft pseudopotentials^29^ using the Quantum-Espresso code suite.^30^ The exchange–correlation part of the energy functional was modeled with the (spin-unpolarized) generalized gradient approximation (GGA), in the PBE parameterization.^31^ Van der Waals interactions were accounted for by use of the vdW-DF functional.^32^ The Au(111) surface was represented by a three-layer slab with the two bottom layers kept fixed during geometry optimization. A periodically repeating, 8 × 4√3 surface cell was used in the lateral direction with a vacuum region of about 19 Å between periodic images. A kinetic energy cutoff of 25 Ryd along with an augmentation charge cutoff of 250 Ryd was used in the truncation of the plane wave expansion. The Brillouin zone integrations were conducted at the *Γ* point. The geometry optimizations were conducted using the BFGS algorithm.^33–36^ Guided by the experimental STM images, the TPB molecule was placed on the surface in three possible geometries (see Fig. S1 in ESI†). In the second step, core excitation energies and oscillator strengths were calculated by the molecular quantum chemistry Amsterdam Density Functional (ADF) code.^37,38^ The electronic structures of the finite systems were obtained by scalar relativistic (SR) self-consistent field (SCF) Kohn–Sham (KS) calculations using the gradient corrected Perdew–Wang exchange functional^39^ and the Perdew correlation functional.^40^ Slater-Type Orbital (STO) functions were taken from the ADF database. The even tempered QZ3P-3DIF basis set was employed to describe the core-excited B and O atoms, while a TZP basis set was used for the remaining atoms of the adsorbed TPB. In the calculation of the B K-edge spectra, core orbitals of the B and O atoms of TPB not involved in the core excitation were treated by the Frozen Core (FC) technique (TZP.1s basis set). A similar FC procedure (but restricted only to the O atoms of TPB not involved in the core excitation) was applied in the calculation of the O 1s excitation spectra. As concerns the surface cluster, the Au atoms directly interacting with the core-excited B (O) atom of the TPB were described with a ZORA Triple Zeta Polarized basis set (TZP), with a FC up to 4f (TZP.4f); a ZORA DZ.4f basis set was used for all the remaining Au atoms of the cluster. The DFT transition potential (TP) method^41^ was employed for the calculations of the O 1s and B 1s core excitation spectra in order to include most of the relaxation effects following the core hole formation. To calculate the Ionization Potentials (IPs), the energy of the 1s^–1^ ionic state is obtained from an unrestricted KS calculation. The TP excitation energies are then shifted with respect to the ΔIP value corresponding to [*ε*_1s_ – ΔKS] (ΔKS stands for ΔSCF Kohn–Sham). All the calculated spectral profiles have been convoluted with Gaussian functions of appropriate Full Width at Half Maximum (FWHM), which has been chosen as yielding the best fit to the experimental spectra. The same computational protocol is used for the calculation of B 1s and O 1s NEXAFS spectra of the free TPB molecule. The geometry of the gas-phase TPB is optimized at the LDA (VWN) potential and TZP basis set starting from the experimental crystal data^42^ without symmetry constraints. The present computational methodology was previously applied to organic molecules on Si(100)^43,44^ and Au(111) surfaces,^45,46^ proving its reliability for describing the polarized K-shell spectra of the adsorbed molecules.

## 3 Results and discussion

### 3.1 Chemistry and morphology

The bottom left panel of Fig. 1 shows the STM image of the monolayer, as obtained by depositing the PBA molecules on the sample at room temperature (RT). We verified that this stage represents the saturation coverage for growth at RT, as the formation of a second layer is inhibited. The surface is characterized by the presence of trilobate structures, assembled in an irregular compact phase. The shape and the dimensions of the structures suggest that they comprise three PBA molecules assembled into TPB, the result of the boroxine condensation process, as depicted in the upper scheme of Fig. 1. The same structures are observed also upon the deposition at lower sample temperatures (*T*_s_ ∼ 200 K). The TPB-covered surface is stable upon annealing at up to 450 K, while at higher temperatures desorption of TPB and formation of larger structures following further polymerization processes are observed (right panels of Fig. 1). The STM image of a 1.5 layer coverage film, obtained by deposition of PBA at *T*_s_ ∼ 200 K, is reported in the central bottom panel of Fig. 1. The molecules of the second layer are still organized as trilobate structures, but their size is larger than for the TPB in the first layer (still visible in the right bottom panel of Fig. 1).

**Fig. 2:**
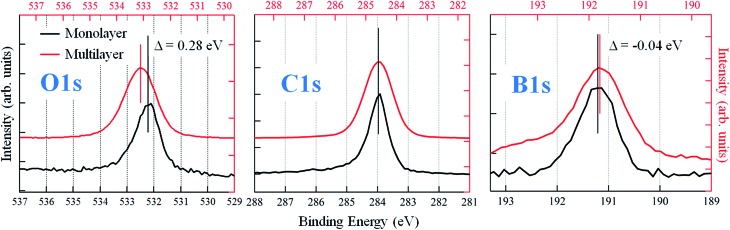
O 1s, C 1s and B 1s X-ray photoelectron spectra of the multilayer (estimated thickness: 55 Å) and monolayer phases. The binding energies of the peaks have been obtained by fitting the curves with Gaussian functions. The energy scales (top and bottom axes) have been rigidly shifted by 0.6 eV in order to align the C 1s signal of the two films. Both the different values of the residual shift observed for O 1s and B 1s in the monolayer and a change in the O 1s/B 1s intensity ratio can be explained with the presence of boroxinated molecules at the organo-metallic interface.

We therefore assign the structures on the second layer to trimers of PBA molecules involved in a hydrogen bonding scheme, as represented in the upper panel of Fig. 1. We verified that thermal annealing at RT of multilayer films grown at low *T*, allows for desorbing all but the monolayer molecular structures. The low temperature boroxination scenario we report here is somewhat different from what was observed for a similar molecule, 1,4-benzenediboronic acid (BDBA), which was found to undergo condensation on Au(111) only upon high temperature annealing or deposition at *T*_s_ ∼ 420 K.^20^ BDBA differs from PBA with a second boronic termination in the *para* position, which promotes the assembly in a head-to-tail configuration, with the formation of hydrogen bonds between boronic terminations of adjacent molecules. The resulting compact hydrogen bond phase hinders the formation of boroxine polymers due to sterical constraints.^11^ Here instead, the PBA molecules are free to assemble in the hydrogen bonding network represented in Fig. 1. For the first layer molecules that interact with the gold surface this assembling scheme represents the precursor of the condensation process, leading to the synthesis of TPB. In general, this indicates the primary role of the morphology of the intermediate phase in the synthesis of a 2D covalent architecture. The chemical composition of the monolayer is confirmed by XPS measurements. Fig. 2 reports the measured XPS O 1s, C 1s and B 1s signals taken of a multilayer grown at *T*_s_ = 200 K (estimated thickness: 55 Å) and of the monolayer obtained by annealing the multilayer at 380 K. We notice first of all that in the PBA network we propose for the multilayer, oxygen atoms are not chemically equivalent any more because of the hydrogen bonds they are involved in. This should in principle be evidenced by the O 1s XPS signal,^47^ where however a single peak is detected. Indeed, an increase of 0.2 eV in its FWHM with respect to the monolayer spectrum is observed, which can be possibly due to the presence of two unresolved components. In the graphs, the binding energy scales relative to the two systems are reported on the top and bottom axes, respectively. Assuming that the interaction of phenyl rings with the substrate is weak, a relative shift of 0.6 eV between the two scales has been introduced in order to align the C 1s peaks of the two films, found at different positions because of the different final state screening by the substrate. As evidenced in the figure, after the screening alignment, a minor additional energy shift (–0.04 eV) of the monolayer with respect to the multilayer remains for the B 1s peak. On the contrary, the O 1s peak displays a positive shift of about +0.3 eV. Moreover, from the analysis of the peak intensities, which takes into account respective cross sections, a sizeable difference in the element stoichiometry is found for the two coverages. In particular, the monolayer is characterized by equal population of O and B atoms whereas the O 1s : B 1s intensity ratio doubles in the multilayer. These findings confirm that, whereas PBA molecules are intact in the multilayer, for the monolayer they condense into TPB molecules, with the loss of one oxygen atom per boronic group in the reaction, due to the formation of a H_2_O molecule. As evidenced in Fig. 2, the formation of boroxine differently affects the O 1s and B 1s binding energies and this was already pointed out for the BDBA condensation, where it was shown that the largest binding energy shift is measured for the O 1s peak.^13^ To further verify this conclusion, we calculated, by means of DFT, the IPs of O 1s, C 1s and B 1s electrons, which were found to be, respectively, 539.83 eV, 290.63 eV and 197.04 eV for PBA and 539.07 eV, 290.43 eV and 196.81 eV for TPB isolated molecules. The trend in the IP of O 1s and B 1s with respect to the C 1s IPs for the two molecules is consistent with the experimental observations reported in Fig. 2.

### 3.2 Electronic properties: NEXAFS and DFT calculations

**Fig. 3:**
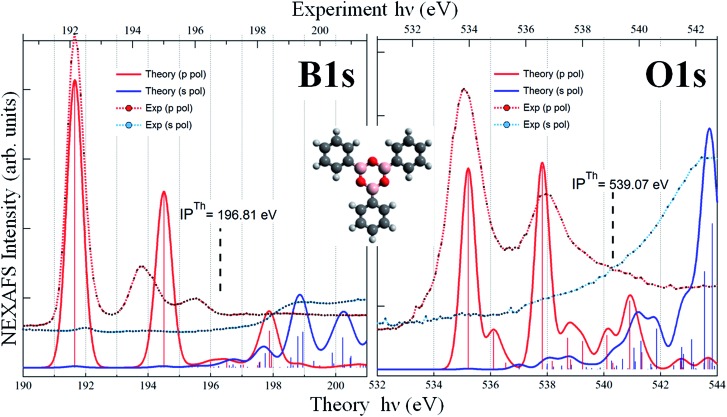
B 1s (left) and O 1s (right) NEXAFS spectra of the TPB as calculated (free molecule) and measured (monolayer) at the two different polarization angles. Bottom and top axes report the theoretical and experimental photon energy scales respectively.

We performed DFT calculations of the TPB molecule, both in its gas phase and adsorbed on the Au(111) surface. As we will discuss later, the main features of the spectra are not affected by the presence of the substrate, indicating that the molecule–gold interaction is weak. We discuss first the results obtained from the calculations of the free molecule, to assign the measured peaks to specific electronic transitions. Since the optimized geometry of the gas-phase TPB molecule is planar, the B (O) atoms of the boroxine ring are equivalent, therefore we discuss only the total B 1s (O 1s) NEXAFS spectra, obtained by summing up the different spectral contributions of the three equivalent atoms for each element. The s- and p-pol spectra have been obtained considering the transitions occurring in the boroxine *xy* plane and in the *z* direction, respectively. The two main peaks characterizing the calculated B 1s p-pol spectrum (at 191.70 and 194.56 eV) derive from two transitions π* MOs mainly originating from the B 2p_*z*_ atomic component of the boroxine ring and from the C 2p_*z*_ components of the phenyl ring directly bonded to the B where the 1s vacancy is created. The higher intensity of the first peak, with respect to the second one, maps the larger B 2p_*z*_ contribution to the final orbital (LUMO for the first transition) and the more diffuse character of the final MO of the second transition. These two peaks are suppressed in the s-pol spectrum, which is featureless in the region below the threshold, well reproducing the dichroic effects also observed in the experimental spectrum. The calculations foresee the presence of features in the B 1s s-pol spectrum only above the ionization threshold; since the final virtual orbitals above the edge are unbound and the LCAO-MO approach in a finite basis set is not able to properly describe them we do not discuss the transitions calculated in this energy region. The dichroic effects observed in the B 1s spectra of the free TPB are present also in the O 1s spectra (right graph of Fig. 3), which show several transitions of mainly π* character for the p-pol spectrum in the region below the threshold, losing their intensity in the s-pol spectrum. In detail, the first two transitions (at 535.2 and 536.1 eV) are towards two π* valence orbitals delocalized over the boroxine and all the phenyl rings with significant 2p_*z*_ contribution from C and B atoms and, to a lesser extent, from the O atoms. Their different intensity maps the decrease of the O 2p_*z*_ component in the final state of the second transition. The structure around 538 eV results from a stronger transition to an orbital mainly localized on the boroxine ring while the following less intense transitions below the threshold have a more diffuse character with contributions of Rydberg O *n*p orbitals. The calculated O 1s s-pol spectrum is almost featureless below the threshold and the structures gain intensity only several eV above the ionization threshold. The comparison between theoretical results of the free TPB and the flat phase experimental data is overall satisfying, as revealed by an analysis of Fig. 3. However, significant overestimation of the energy separation between the two calculated main peaks is evident for the B 1s p-pol spectrum, while for O 1s the energy shifts among the experimental absorption features are well reproduced by the calculations. This overestimation could be tentatively associated with a possible interaction of the TPB with the Au surface; however, it is also present in the calculated B 1s p-pol spectrum of the TPB@Au(111) model cluster.

Therefore, it appears that the ability of DFT-TP to describe the more intense core level excitations deteriorates when the core hole is localized on boron compared to oxygen. To investigate the underlying reasons for disagreement with the experiment, we have performed other simulations of the B 1s spectrum of the free TPB, employing different computational schemes both in the DFT and in the Time Dependent DFT (TDDFT) frameworks. Since all calculations confirm the same nature of the two peaks we are confident about the proposed assignment of the measured B 1s spectral features; the observed discrepancies have to be ascribed to the limited amount of final state correlation effects that are included in computational approaches, which deserve future investigation.

### 3.3 Boroxine–Au(111) interaction: evidence of orbital coupling and an ultra-fast charge delocalization channel

The role of the molecule–substrate interaction in determining the electronic properties of the system has been investigated by performing DFT calculations of the NEXAFS transitions in the TPB molecule adsorbed on the Au(111) surface. Inspection of the STM images of the monolayer (Fig. 1) reveals that (i) the average lateral distance between adsorbates is of the order of 7–8 Å, suggesting that adsorbate–adsorbate lateral interactions are rather weak; (ii) the adsorption pattern is such that a line passing through the centers of two phenyl rings makes an angle of about 19° with respect to the high-symmetry [112] and [110] directions of the Au(111) surface. Accordingly, we considered three different adsorption models for TPB on the Au(111) surface with the boroxine ring centered on a high-symmetry site of the Au surface (on top, hcp or fcc), and the TPB moiety being rotated by about 19° with respect to the high symmetry [110] direction. Interestingly, once the center of the boroxine ring of TPB is placed on a high symmetry site, the centers of the phenyl rings almost exactly coincide with other high-symmetry sites of the gold surface (see Fig. S1 of ESI†). In particular, when the boroxine ring is centered on a fcc (on-top) site, the centers of the three phenyls are close to on-top (hcp) positions. On the other hand, if we place the center of the boroxine ring on a hcp site, the phenyls are centered on fcc sites. All three adsorption models were tested, placing a single TPB molecule in a supercell where the Au(111) surface was represented by a three-layer slab while a 
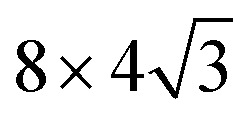
 surface cell was used in the lateral direction.

**Fig. 4:**
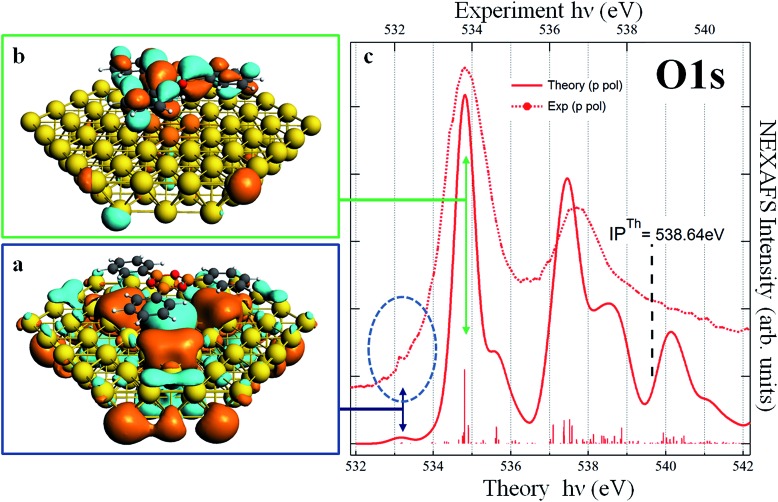
TPB@Au(111). Panels (a) and (b): plot of the calculated DFT-TP final state involved in the O 1s transition at 533.2 eV and 534.8 eV respectively. Panel (c): comparison of the experimental (already shown in Fig. 3, right panel) and calculated p-pol O 1s NEXAFS spectra.

**Fig. 5:**
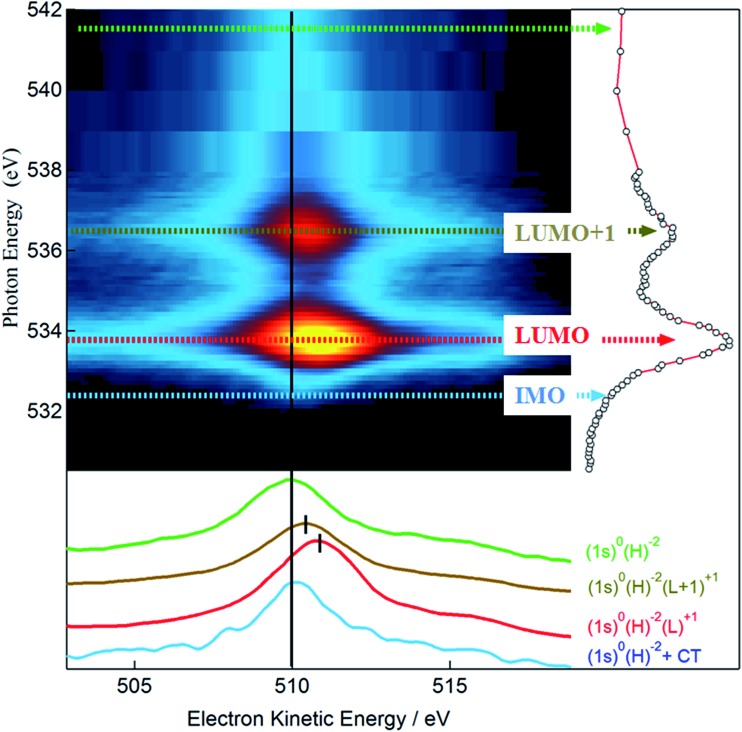
Oxygen K-edge RAES map of the TPB monolayer. RAES color intensity is shown as a function of photon energy and electron kinetic energy. The corresponding NEXAFS spectrum is shown alongside. Colored, dotted arrows indicate photon energies where single RAES spectra, displayed in the bottom panel, have been measured. The electronic configuration of the final states is indicated for each resonant scan, with the labels reporting the electronic balance in the core level (1s), valence band (H) and LUMO orbital (L) involved in the transition. The IMO Auger line shows no spectator shift, consistent with ultrafast charge transfer to the Au substrate from this orbital.

For all starting geometries, during structural optimization, the TPB adsorbate does not appreciably change its initial orientation with respect to the [110] direction, while slightly relaxing its planarity due to the three phenyl rings pointing towards the gold substrate. The final relaxed configurations for these models are shown in Fig. S1 of ESI.† The optimized on-top and hcp adsorption geometries are characterized by Au–B vertical distances in the ranges 3.58–3.61 Å and 3.60–3.62 Å, respectively, while slightly larger vertical distances in the range 3.74–3.75 Å are observed in the fcc adsorption geometry. The structural data all point towards a weak interaction (physisorption) between TPB and the Au(111) substrate. Theoretical simulations of the B 1s NEXAFS spectra have been performed on all three adsorption cluster models cut out from the optimized periodic structures. No significant differences have been found among the three configurations and all the spectra closely resemble those calculated for the free molecule (see Fig. S2 of ESI†). Since the assignment of the calculated spectral features is similar for the three adsorption models, a detailed discussion will be done for the fcc cluster model only (shown in Fig. S3 of ESI†). The dominant spectral features continue to involve final states of well defined molecular character; the effect of the Au–TPB interaction can be related to the relatively small intensity redistribution among multiple closely spaced transitions around the main B 1s → π* excitation, characteristic of the isolated molecule. The detailed analysis of the spectra as well as the description of specific orbitals involved in the NEXAFS transitions can be found in the ESI† and both point to an extremely weak, Au induced perturbation of the chemical environment around boron atoms. Quite interestingly, the analysis of the chemistry and of the coupling of the oxygen atoms reveals well defined and pronounced fingerprints of the molecule–substrate interaction. Panel (c) of Fig. 4 reports the calculated p-pol O 1s NEXAFS spectrum of the TPB@Au(111) system in the fcc adsorption geometry, together with the corresponding experimental curve. By comparison with the free molecule calculated spectrum shown in Fig. 3, we observe that the two main bands at ∼535 eV and ∼537.5 eV no longer originate from single isolated transitions but rather, as discussed below, from several distributed transitions. The first band, at 534.8 eV, involves a π* final state orbital with Atomic Orbital (AO) contributions from B, C, and O atoms and it is delocalized over all the TPB skeleton (panel b of Fig. 4). Transitions with lower intensity, within 0.1 eV of the main line, involve final states with predominant Au AO contributions, together with a smaller weight of O 2p_*z*_. Comparison with the polarized O 1s spectrum relative to the free TPB reveals the signature of the Au–TPB interaction. In particular, while in the absence of the Au support the intensity was concentrated in a single principal line, the molecule–gold interaction promotes a partial redistribution of the intensity to several closely spaced levels, with varying weights of Au AO contributions. The most intense transition, giving rise to the shoulder visible at 535.6 eV in the computed spectrum, involves again a predominantly π* molecular final state, delocalized over the entire molecule, with a small degree of Rydberg character, and with contributions from the AOs of the Au atoms. The second absorption band at 537.5 eV results from several closely spaced O 1s transitions involving contributions of diffuse O AOs to the final MOs. In particular, the final state of the most intense transition at 537.5 eV, of mixed valence-Rydberg nature, also contains contributions from AOs of the gold atoms mixed with C 2p atomic components of the π*(C=C) orbitals of the phenyl rings, and 2p_*z*_ AOs of B. However, the most interesting effect of the molecule–gold interaction in the calculated spectrum is represented by the occurrence of a weak spectral feature at 533.2 eV, just below the principal line of the isolated molecule. The corresponding transitions involve a final state with strong Au AO contributions, shown in panel (a) of Fig. 4. The charge distribution revealed by the depicted orbital suggests that the nature of the TPB–Au interaction exhibits stronger character than a mere physisorption. Although we cannot identify a clear peak in the experimental NEXAFS as belonging to the Au-hybridized MOs of the coupled TPB, we can nevertheless observe a significant broadening of the resonance at 534 eV with respect to the main B 1s transitions, with an intensity increase in the low energy tail of the peak. This spectral region is highlighted by the dashed circle in Fig. 4, and evidences an increased spectral weight, possibly affected by the transition to the interface hybrid orbital depicted in the panel (a) of Fig. 4. In order to clarify the nature of this spectral feature in the NEXAFS, revealing its relation with the electronic coupling between the substrate and the oxygen of the boroxine ring of the adsorbed TPB molecules, we examined the resonant photoemission at the O K-edge. RAES makes use of the competition of the inner shell core-hole lifetime (for O 1s *τ*_ch_ = 4 fs (ref. 48)) and delocalization of the photoexcited electrons.^49,50^ From the branching of the competing core-hole decay channels, we study the delocalization dynamics of excited electrons over empty molecular orbitals. Specifically, we acquired a series of valence band photoemission spectra over an extended kinetic energy range, with photon energies spanning across the O 1s absorption edge. For the sake of simplicity we indicate hereby the main resonant bands of the O 1s NEXAFS as LUMO (Lowest Unoccupied Molecular Orbital) and LUMO+1 and the region corresponding to the theoretically calculated Interface Molecular Orbital as IMO. The RAES series of spectra is shown in Fig. 5 as a 2D intensity color map as a function of both photon and kinetic energy, *I*(*hν*, *E*_k_). The non-resonant part of the photoemission spectra as measured in the pre-resonant region has been subtracted from each spectrum in the map. We follow the spectral position of the main Auger line, which sets in at the O 1s → LUMO resonance (*hν* = 533.7 eV). While for photon energies above the ionization threshold (*hν* > 538 eV) the position of the Auger line is constant (*E*_k_ = 510 eV), reflecting the final state configuration of the molecule (2 holes in the VB), the Auger peak position at the LUMO and LUMO+1 resonances (at *hν* = 533.7 and 536.7 eV) reflects the presence of a localized spectating electron excited from the 1s core level. Here the final configuration is effectively a single hole in the valence band (*i.e.* 2 holes in VB, 1 electron in LUMO or LUMO+1) and the resonant Auger peak is shifted to higher kinetic energies due to additional Coulomb potential of the spectator electron. If on the other hand the final state orbital is efficiently coupled to a much larger system, the core-excited electron may delocalize away prior to the core-hole decay, and the resulting Auger peak is found with no spectator shift.^50^ Detection of this spectator-shifted component in the resonant Auger spectrum highlights the localization of the core-excited electron in the empty orbital of the uncoupled molecule. Fast delocalization of the core-excited electron, on the other hand, is a strong evidence of orbital coupling. The lower panel of Fig. 5 compares single RAES spectra measured in correspondence of the IMO, LUMO, and LUMO+1, together with the Auger peak measured above the ionization edge. Indeed, we find that the oxygen Auger peak at the LUMO and LUMO+1 resonances are spectator shifted (by ∼0.9 eV in the former case), indicating a poor orbital coupling with the substrate. On the contrary, the resonant component at lower photon energies (*hν* = 532.3 eV) addresses the delocalization dynamics from the IMO that stems from the orbital coupling with the Au substrate. In fact, the corresponding Auger peak aligns at 510 eV, as with the Auger peak measured above the edge. This confirms that the additional empty state spread over oxygen atoms is very well coupled with the Au substrate and provides a pathway for ultrafast transfer of excited electrons over empty interfacial orbitals.

## 4 Conclusions

In conclusion, we have presented a combined theoretical and experimental study of the electronic properties of the boroxine–gold interface. The boroxination of PBA trimers into TPB molecules has been characterized by means of both STM and XPS. The electronic structure of the TPB@Au(111) system has been described by means of DFT-based calculations and NEXAFS measurements. A detailed inspection of the MOs involved in the transitions at the O K-edge revealed the presence of an unoccupied orbital strongly hybridized with gold atoms, close to the Fermi level. The nature of this state is corroborated by the O 1s RAES, which evidences strong coupling of these orbitals with the Au substrate, allowing for ultrafast delocalization of core-excited electrons. Our findings shed light on the chemistry of the boroxine–metal interfaces and demonstrate that the boroxine group, even on weakly reactive surfaces, represents a promising building block for the functionalization of electrodes in organic electronics. 2D boroxine COFs can therefore be not only considered as mere templates, but as having an active role in tailoring the electronic transport between the guest molecules and the substrate.

## Author contributions

All authors contributed to the manuscript. A. C., G. F., D. T., M. Sten., G. C., D. T., D. C., G. K. contributed to the writing of the manuscript; D. T., G. F., G. B., S. F., M. Sten., H. U. performed the calculations, G. F., D. T. analyzed the theoretical findings; A. C., M. Str., A. V., D. C., G. K. performed the X-ray spectroscopy measurements; Z. F., C. D. performed and analyzed STM measurements; M. Str. analyzed the XPS and NEXAFS data, D. C. and A. M. analyzed and discussed RAES data; A. C., G. F. conceived the experiments and the theoretical approach.
