# Supplementary material for: Electronic properties of the boroxine–gold interface: evidence of ultra-fast charge delocalization
Source: Chem Sci. 2017 Mar 8;8(5):3789–98. doi: 10.1039/c6sc05632f (PMC5436552; doi:10.1039/c6sc05632f)
Supplement: Supplementary file 1 [file SC-008-C6SC05632F-s001.pdf]

Electronic Supplementary Material (ESI) for Chemical Science.
This journal is © The Royal Society of Chemistry 2017

Electronic properties of the boroxine-gold interface: evidence of ultra-fast charge delocalization.

Daniele Toffoli[§], Matus Stredansky[¥,ǂ], Zhijing Feng[¥,ǂ], Gabriele Balducci[§], Sara Furlan[§], Mauro Stener[§], Hande Ustunel[ȝ], Dean Cvetko[ǂ, ʃ], Gregor Kladnik[ǂ, ʃ], Alberto Verdini[ǂ], Alberto Morgante[¥,ǂ], Carlo Dri[¥,ǂ], Giovanni Comelli[¥,ǂ], Giovanna Fronzoni[§,], Albano Cossaro[ǂ,*]*

¥ Department of Physics, University of Trieste, via A. Valerio 2, 34127, Trieste, Italy

ǂ CNR-IOM Laboratorio Nazionale TASC, Basovizza SS-14, km 163.5, I34012 Trieste, Italy

§ Department of Chemical and Pharmaceutical Sciences, University of Trieste, via L. Giorgieri 1, 34127, Trieste, Italy

ʃ Faculty of mathematics and physics, University of Ljubljana, Ljubljana, Slovenia

ȝ Department of Physics, Middle East Technical University, 06531 Ankara, Turkey

Supporting Information

1. Comparison of the TPB@Au(111) B(1s) NEXAFS spectra of the fcc, hcp and On-Top model systems.

Guided by the experimental STM images, the TPB molecule was placed on the surface in three possible geometries. To generate these geometries, the base of the triangle formed by the

three phenyl rings was aligned with the $[\bar{1}10]$ direction, the boroxine ring was positioned a distance of about 3 Å above the fcc, hcp and on-top (the center of the boroxine ring directly above a Au atom on the top layer of the slab) symmetry sites and finally the molecule was rotated counterclockwise by about 19.1°. The optimized geometries are displayed in Figure S1.

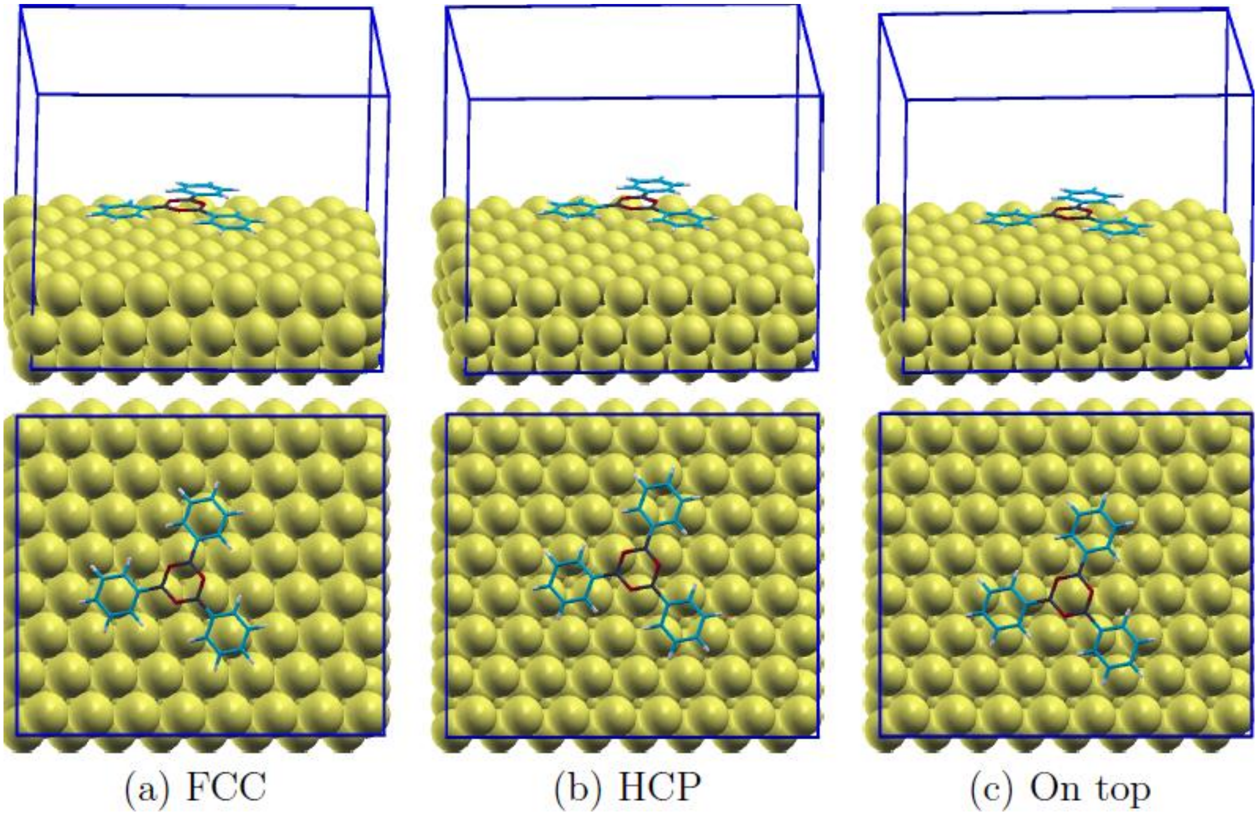

Figure S1. Side (upper panels)and top (lower panels) views of the three adsorption geometries of TPB on Au(111): fcc (a), hcp (b) and on-top (c).

The calculated B(1s) NEXAFS spectra of the TPB@Au(111) model systems are reported in Figure S2. As can be seen from an inspection of the computed absorption profiles, there are no major qualitative differences among the three spectra for both perpendicular (p-pol), and parallel (s-pol) polarizations. Actually the s-pol spectra show only a rise in intensity near the B(1s) thresholds, and small differences among the NEXAFS spectra computed for the three models are visible only in the p-pol case. These differences consist of small relative shifts on the energy scale which is appreciable only in the case of the hcp model, and a small spectral feature visible around 193 eV which is only present in the On-Top model system. Computed B(1s) TS and ΔSCF IP potentials do not show significant variations among the three TPB adsorption models, as confirmed by the corresponding values reported in Table S1:

Table S1. DFT B(1s) TS and ΔSCF IP potentials for the three TPB@Au(111) adsorption models. Reported values are in eV.

Model	B1(TS)	B1(ΔSCF)	B2(TS)	B2(ΔSCF)	B3(TS)	B3(ΔSCF)
FCC	202.66	196.55	202.66	196.55	202.66	196.55
HCP	202.73	196.55	202.74	196.54	202.74	196.55
On-Top	202.80	196.67	202.79	196.66	202.79	196.66

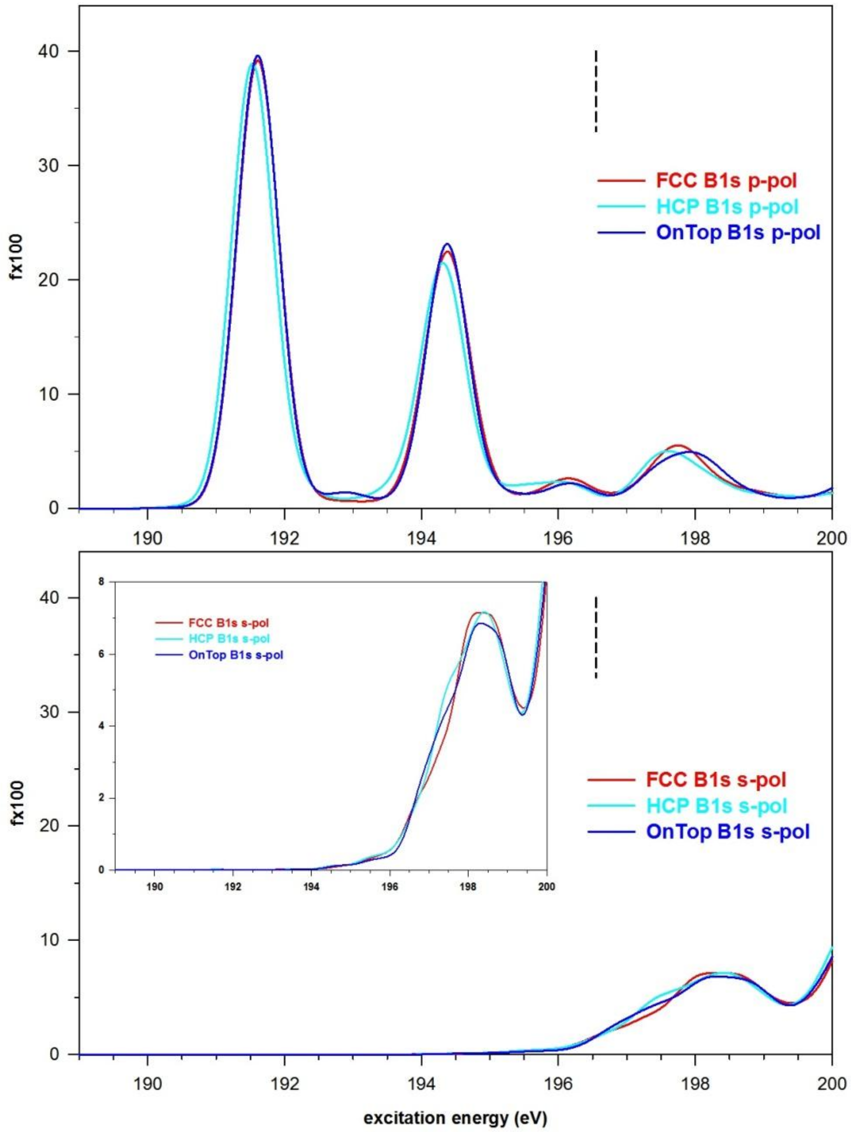

Figure S2. Calculated NEXAFS B(1s)profiles for the three adsorption models of TPB on Au(111). Upper panel: p-pol. Lower panel: s-pol.

Since the assignment of the calculated spectral features is similar for the three adsorption models, a detailed assignment will be done for the fcc cluster model only (shown in Fig. S3).

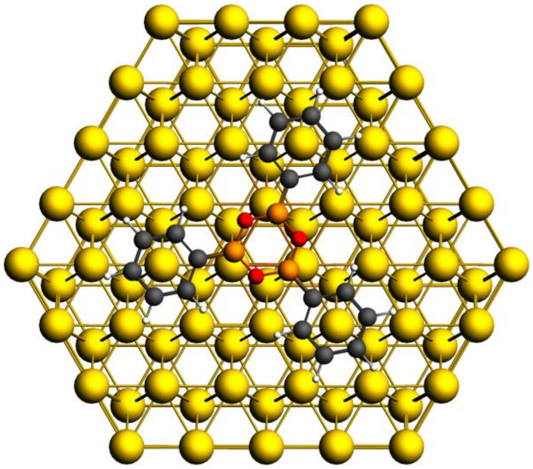

Figure S3. View of the fcc model cluster of TPB on Au(111): B atoms : orange; O atoms :red; C atoms : dark gray; [Au_{84}] surface cluster : gold

2. Effects of the TPB-gold interaction on B1s NEXAFS

The experimental and calculated B1s polarized spectra for the fcc adsorption geometry of TPB@Au(111) are reported in Figure S4; to facilitate the comparison between experiment and theory, the theoretical spectra have been shifted on the experimental energy scale (see figure captions).

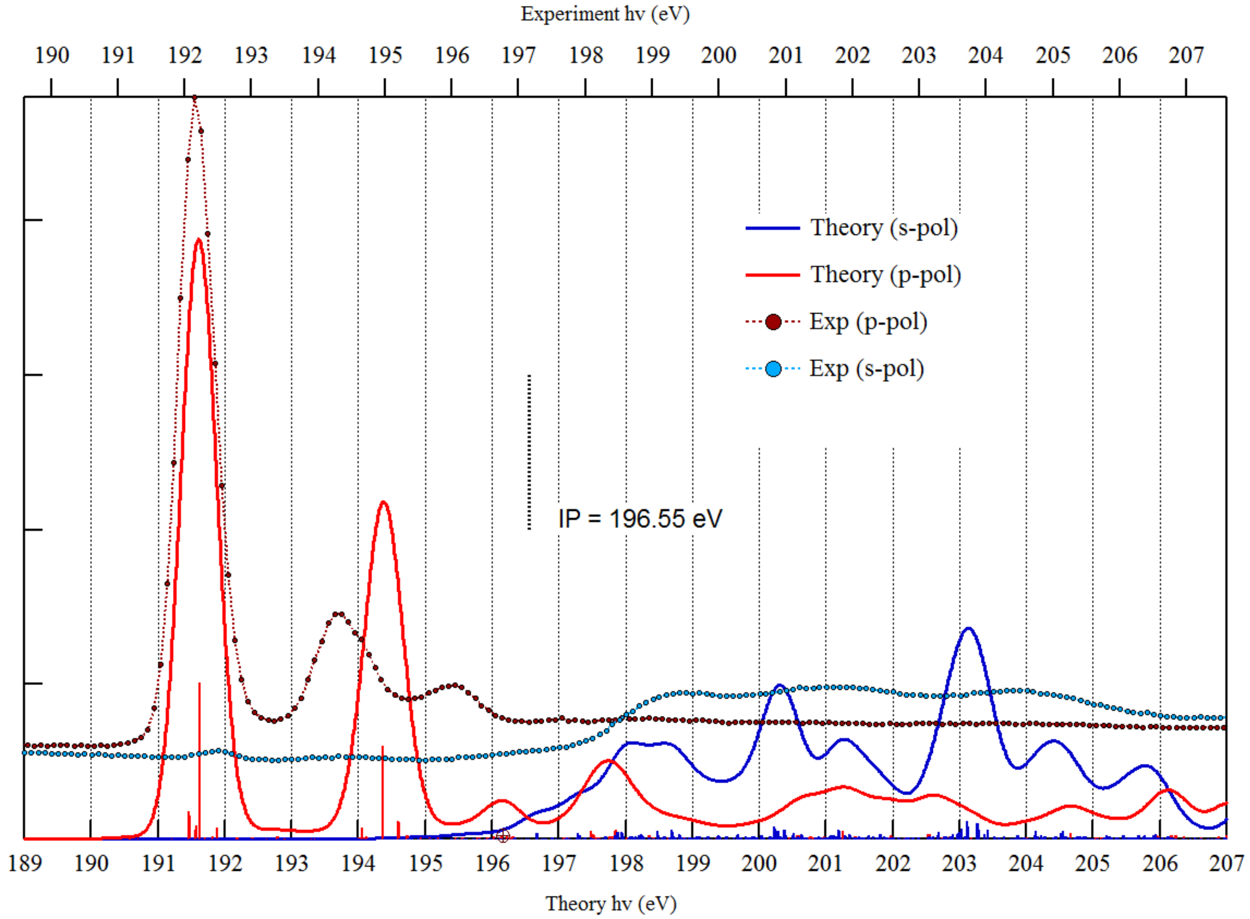

Figure S4. Experimental and calculated polarized B 1*s* NEXAFS spectra of TPB@Au(111) in the fcc adsorption geometry. A rigid shift of 0.5 eV to higher excitation energies has been applied to the calculated profiles to align the energy position of the first absorption peak to the experimentally-derived value.

The calculated s-pol spectrum is featureless for excitation energies below threshold, in agreement with the experiment. The above-threshold experimental absorption intensity rises to an almost constant value. However, since our computational procedure does not properly describe unbound states, we will not discuss the calculated data in this energy region. The experimental p-pol spectrum is instead characterized by a strong absorption peak centered at 192.15 eV, followed by a broader feature with a maximum at 194.25 eV, and by another weaker signal centered at 196.05 eV. The above-threshold portion of the experimental p-pol spectrum is instead featureless. The calculated p-pol spectrum agrees qualitatively with the experiment concerning both the energy position of the absorption features and intensity distribution. On a more quantitative level, there are discrepancies. We proceed with an assignment of the spectral features to specific single-particle excitations. The first peak in the theoretical p-pol spectrum derives its intensity from several transitions among which the more intense, centered at 191.6 eV, corresponds to a final orbital of π^* character, mainly localized on the boroxine ring and the phenyl ring directly bonded

to the B atom where the 1s vacancy is created. A plot of this virtual orbital, of marked molecular character, is reported in the left panel of Figure S5. The stronger intensity of this transition, compared with the second most intense one calculated at 191.4 eV (whose final state presents also contributions from Au atomic orbitals), is related to the larger weight of the B 2p_z orbital in the final state. The calculated absorption peak with a maximum at around 194.5 eV derives most of its intensity from two transitions, a stronger one at 194.4 eV and a weaker one at 194.6 eV respectively, to final states of marked molecular π character. Both final states, similarly to those involved in the first absorption peak, extend through the boroxine ring and the phenyl ring bonded to the B atom with the core vacancy, but display a higher degree of delocalization on the remaining phenyl rings of TPB. Plot of the state involved in the transition at 194.4 eV is included in the right panel of Figure S5.

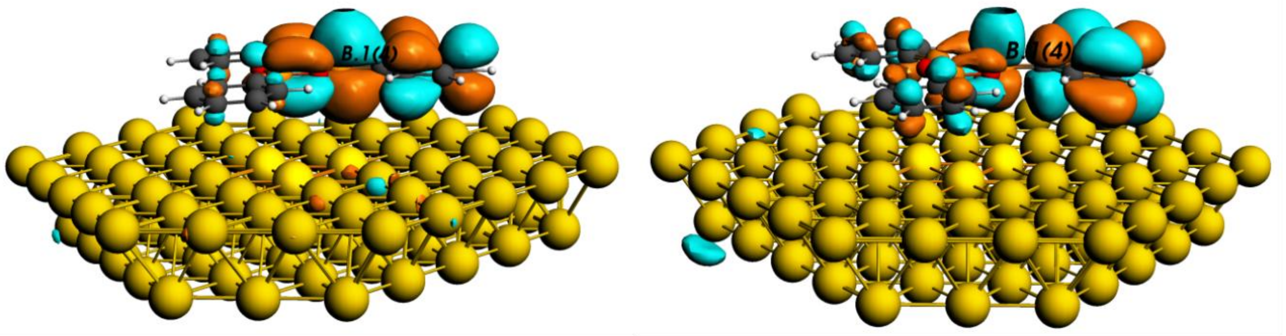

Figure S5. *Left*: plot of the final state involved in the transition at 191.6 eV. *Right*: plot of the final state involved in the transition at 194.4 eV.

A third, less intense feature calculated at 196.2 eV, in close proximity to the B 1s threshold involves a large number of transitions with low intensity the most prominent of which fall in the energy interval 195.9-196.4 eV to highly delocalized final states with non-negligible AO contributions of the Au atoms of the surface. We conclude that the dominant spectral features still involve final states of molecular character; the effect of the Au-TPB interaction can be related to the relatively small intensity redistribution to several closely spaced transitions around the dominant B1s$\rightarrow\pi^*$ excitation, characteristic of the isolated molecule. Furthermore, the observation of a weak Au-TPB interaction agrees with the strong dichroic effect, which is experimentally detected as a consequence of the preferred flat adsorption geometry of TPB, indicating thus only little perturbation of the flat equilibrium gas-phase geometry of the adsorbate.
